# A Key Pathway to Cancer Resilience: The Role of Autophagy in Glioblastomas

**DOI:** 10.3389/fonc.2021.652133

**Published:** 2021-06-10

**Authors:** Elisa Helena Farias Jandrey, Marcelle Bezerra, Lilian Tiemi Inoue, Frank B. Furnari, Anamaria Aranha Camargo, Érico Tosoni Costa

**Affiliations:** ^1^ Molecular Oncology Center, Hospital Sírio-Libanês, São Paulo, Brazil; ^2^ Ludwig Institute for Cancer Research, University of California San Diego (UCSD), San Diego, CA, United States

**Keywords:** autophagy, glioblastoma, intratumoral heterogeneity (ITH), drug resistance, cell invasion, pro-tumoral

## Abstract

There are no effective strategies for the successful treatment of glioblastomas (GBM). Current therapeutic modalities effectively target bulk tumor cells but leave behind marginal GBM cells that escape from the surgical margins and radiotherapy field, exhibiting high migratory phenotype and resistance to all available anti-glioma therapies. Drug resistance is mostly driven by tumor cell plasticity: a concept associated with reactivating transcriptional programs in response to adverse and dynamic conditions from the tumor microenvironment. Autophagy, or “self-eating”, pathway is an emerging target for cancer therapy and has been regarded as one of the key drivers of cell plasticity in response to energy demanding stress conditions. Many studies shed light on the importance of autophagy as an adaptive mechanism, protecting GBM cells from unfavorable conditions, while others recognize that autophagy can kill those cells by triggering a non-apoptotic cell death program, called ‘autophagy cell death’ (ACD). In this review, we carefully analyzed literature data and conclude that there is no clear evidence indicating the presence of ACD under pathophysiological settings in GBM disease. It seems to be exclusively induced by excessive (supra-physiological) stress signals, mostly from *in vitro* cell culture studies. Instead, pre-clinical and clinical data indicate that autophagy is an emblematic example of the ‘dark-side’ of a rescue pathway that contributes profoundly to a pro-tumoral adaptive response. From a standpoint of treating the real human disease, only combinatorial therapy targeting autophagy with cytotoxic drugs in the adjuvant setting for GBM patients, associated with the development of less toxic and more specific autophagy inhibitors, may inhibit adaptive response and enhance the sensibility of glioma cells to conventional therapies.

## Introduction

Glioblastoma (GBM, grade IV astrocytoma) is the most frequent, life-threatening malignant brain tumor and one of the most resilient of all human malignancies. Those tumors are classified and subtyped based on histopathological traits, clinical presentation, and molecular status (1). The current treatment for GBM includes gross neurosurgical resection with the oral use of alkylating agent temozolomide (TMZ), which is given concurrently with radiotherapy (RT) and as an adjuvant monotherapy. Despite aggressive treatments, patients have a low median survival of ~12 months (2–4).

One of the key factors in GBM’s aggressiveness and resilience is their high cell plasticity: a concept associated with phenotype switching, based on the reactivation of transcriptional programs related to the acquisition stem cell properties and the migratory phenotype (5). In the context of anti-glioma therapies, cell plasticity enables tumor cells to change to a cell phenotypic identity, enabling them to survive the dynamic changes of the tumor microenvironment (TME) and to escape surgery and radiotherapy margins by migration. A remarkable example of this plasticity in GBM cells was conceptualized in the “go-or-grow” dichotomous concept in gliomas. It is based on the notion that phenotypically distinct GBM cells (at the “go” or “grow” states) coexist and cooperate to promote tumor growth and clinical relapse: chemoradiation effectively eliminates the bulk population of highly proliferative cells (at the “grow” state), leaving behind a subpopulation of dormant/migratory cells (at the “go” state). “Go” and “grow” states are completely reversible insofar as GBM cells change their phenotypes without genetic mutations. This plasticity is controlled by different signaling pathways that drive adaptive responses and emerge as a non-genetic source of functional intratumoral heterogeneity that, ultimately, mirror tumor resiliency and high patient mortality (2, 6, 7).

Autophagy (greek “self-eating”) is a good example of signaling pathway associated with the phenotype switching and metabolic flexibility of GBM cells. It is primarily a degradative pathway characterized as a fast route by which damaged cytoplasmic materials (collectively named ‘cargo’) are delivered to the lysosomes for recycling. Autophagy can be categorized into 3 subtypes called, micro-autophagy, macro-autophagy, and chaperone-mediated autophagy (for more detailed insights into the different autophagic pathways see (8–10).

Macroautophagy (hereafter referred to as autophagy) must take place on a baseline in each cell to withdraw damaged and functionless organelles, providing metabolites to synthetic pathways and sustaining energetic homeostasis. In the brain, baseline autophagy is important as a clearance mechanism of disease-related proteins in neurons and also in astrocytes, and autophagy dysfunction may contribute to the progression of neurodegenerative diseases (11). However, a selective activation of autophagy can be observed in various pathophysiological and/or stress situations (12–15). For example, in normal brain Beclin-1 (BECN1), a gene with a central role in autophagy induction (16), was not expressed by neurons or glial cells, but showed strong cytoplasmic overexpression in primary GBM cells (17). Moreover, in response to standard of care in patients with GBM (radio- and chemotherapy), the autophagy pathway is upregulated giving tumor cells an advantage for survival. In a series of clinicopathological studies, cancer cells exhibit an increased autophagy activity linked with poor prognosis and aggressive clinical behavior (17–19). Those are emblematic examples of the ‘dark-side’ of autophagy, acting as a therapy-responsive mechanism associated with a pro-tumoral adaptive response (20–25).

On the other hand, there are numerous reports, mostly from *in vitro* cell-based studies, showing an anti-tumoral function of autophagy. Those reports have clearly shown that excessive activation of the autophagy by prolonged or supraphysiological doses of stress signals, may lead to massive removal of cytosolic material, leading to a specific type of non-apoptotic cell death, named type II programmed cell death, or autophagic cell death (ACD). ACD is characterized by large-scale autophagic vacuolization of the cytoplasm in the absence of chromatin condensation and can be specifically blocked by the inhibition of autophagy-related genes (ATG) (26, 27). Due to this ‘dual’ role in human cancers cells, autophagy is, therefore, often been described metaphorically as a ‘double-edged sword’ in cancers. Importantly, the studies that explore the mechanisms of ACD are mostly from *in vitro* cell-based approaches, which provide us a precious source of mechanistic insights, but are of limited translational relevance. Of note, there is no doubt that GBM cells activate autophagy shortly before or during their death in according to the external cues or internal stimuli received, but it is still controversial whether this activation contributes to cell death or rather represents a last attempt of survival.

So, to understand the real effect of autophagy in GBM disease is necessary to analyze cancer cells under normal pathophysiological conditions and therapeutic doses. In the next sections, we will focus on the specific extracellular signals that surround tumors and play an important role in controlling autophagy in GBM cells. Important is the notion that our particular emphasis was given to studies that evaluate the relationship between autophagy and GBM from a perspective of understanding and treating human disease. Therefore, studies using *in vitro* cell-based models, inducing ACD by excessive stress signals, were not fully considered here, except for the mechanistic data.

## Autophagy Activation as a Response to Pathophysiological Stress

Necrosis and acidic stress are the most important stress signals in GBM microenvironment related with autophagy activation. Tumor necrosis is a histological hallmark of grade IV astrocytic tumors with prevalence in almost 90% of patients with GBM (1, 28, 29). Necrosis appears as either multifocal areas (micronecrosis) or broad necrotic areas surrounded by hyperproliferative zones of tumor cells, called perinecrotic niches (PNN), which is visible as a soft, gray rim surrounding necrotic areas by magnetic resonance imaging (MRI). During disease progression and treatment response, GBM cells have to change their metabolism to survive in PNN, characterized by intermittent hypoxia (defined by low oxygen levels, pO_2_ < 3%) and starvation conditions due to poor functional vasculature (30, 31). This configuration is indirectly linked to poor patient outcome and associated with radio and TMZ resistance (32, 33). Hypoxia, *per se*, is well known to create radiation and chemotherapy resistances (34). As part of the physiological adaptive response, the PNN stimulates the stabilization of hypoxia-induced factors (HIFs), HIF1a and HIF2a, resulting in a driving force for activation of anti-apoptotic and pro-migratory transcriptional programs, supporting angiogenesis (35, 36), and re-expression of markers and properties typical of glioma stem cells (GSCs) (30, 37–42). Interestingly, hypoxia, starvation and conventional anti-glioma therapies stimulate the onset of autophagy above baseline levels in GBM cells.

Hypoxic conditions also shift GBM cells towards aerobic glycolysis, rather than mitochondrial oxidative phosphorylation, promoting an acidic environment, potentially favoring tumor invasion by pH-dependent activation of proteinases (e.g. heparanases and cathepsins) (43). Heparanase (HPSE) is an endo-β-D-glucuronidase that has both enzymatic and non-enzymatic functionalities in a pH-dependent manner. HPSE expression is intrinsically correlated with GBM progression, worse prognosis (44), and cell invasion (45). Intriguingly, autophagy is one of the cellular mechanisms regulated by heparanase activity in various tumors, including brain tumors (46). Notably, autophagy induced by starvation in GBM cells was prevented by the use of a potent heparanase inhibitor. Moreover, in these cells the pro-tumorigenic function of heparanase is mediated by autophagy activation, enhancing chemotherapy resistance in nutrition-stressed environments. The mechanism underlying heparanase-induced autophagy is not fully understood but appears to involve mTOR1 inhibition, which plays a pivotal role in nutrient-sensing and autophagy regulation *in vitro* (47).

Cathepsins belong to a class of cysteine proteinases that is mainly expressed by GSC subpopulations of IDH wild-type GBM patients (48). Cathepsins can be secreted into the extracellular space and have an optimum activity on acidic environments to further activate MMP proenzymes (49), with have an important role in controlling tumor cell invasion, stem cell phenotypes (50–53) and tumor progression (54). Cathepsin D levels, for example, are strongly and positively correlated with LC3A and LC3B expression in GBM patients (markers for autophagosome levels) (17). Moreover, inhibition of Cathepsin D attenuates autophagy, leading to increased radiosensitivity in GBM cells. In radioresistant cells, Cathepsin D has been positively correlated with LC3-II and negatively correlated with p62 (55), a protein that targets specific cargoes for autophagy (56). The expression levels of another member of family, the Cathepsin L, are higher in GBM compared to low-grade gliomas (57), exerting an important role in migratory phenotype (51, 52, 58, 59) and γ-radiation-induced GBM cell invasion (59). Interestingly, autophagy inhibition by trifluoperazine induces radiosensitivity in GBM cells mediated by Cathepsin L downregulation (60).

Interestingly, at PNN (i.e. under physiological hypoxia), autophagy activation via BNIP3/BNIP3L is a survival mechanism that promotes GBM progression and resistance to anticancer therapies *in vivo* (61). Recently, a global analysis conducted by Bronisz et al. that included 41 GBM patient’s cohort identified the autophagy pathway as the unique de-regulated pathway in PNNs of primary GBMs (32). These analyses indicate that poorly perfused tumor regions are likely to have increased baseline autophagic levels and, therefore, under hypoxic conditions, the increased autophagic flux may play an adaptive role (62, 63). Under hypoxia, autophagy is activated by BECN1 phosphorylation *via* the HIF-1a/BECN1 signaling pathway, one of the initial steps in the assembly of autophagosomes from pre-autophagic structures (64–66). Moreover, PNN in GBM disease also show the overexpression of interleukin 6 (IL6), an inflammatory cytokine that is essential for hypoxia-induced autophagy and induction of invasive programs in GBM cells (67–71). At this point, is important to notice that under *in vitro* prolonged hypoxic stress (48-72h, <1% pO_2_), the gene BNIP3 (Bcl-2/adenovirus E1B 19kDa-interacting protein 3), a pro-apoptotic Bcl-2 family member, is upregulated, leading to hypoxia-dependent ACD in GBM cells (72). Mechanistically, BNIP3 upregulation releases BECN1 from the complexes with Bcl-2 or Bcl-xL, allowing BECN1 to activate autophagy (73). It becomes especially critical to note that the nature of the autophagic response to hypoxia - a cytoprotective or cytotoxic output - depends on the extent and duration of the microenvironmental stressor, on the experimental design, as well as, on the genetic background of the tumor cells.

Alternative forms to GBM cells to adapt or to avoid poor oxygenation and hostile microenvironment are through the vasculogenic mimicry (VM) phenomenon (74) and the activation of migratory programs by altering the composition of the TME (75–77). VM represents an impressive example of a higher phenotype flexibility of GBM cells. GBM cells capable of VM formation organize themselves into functional vascular-like structures, ensuring tumor blood supply independently of normal blood vessels or angiogenesis. In this scenario, it has been shown that VM formation in glioma patients was associated with the expression of BECN1 (16).

Moreover, as a part of adaptive programs, VM formation is also promoted by Bevacizumab (BVZ)-induced autophagy in GSC, an anti-VEGF antibody that received accelerated approval by the FDA to treat recurrent GBM (78), which is associated with tumor resistance to antiangiogenic therapy (see below) (79). VM was also associated with high expression of HIF-1α (80) and upregulation of the IL-8/CXCR2 pathway (81). It is also conceivable that autophagy may contribute to the increased production of multiple pro-invasive cytokines, including interleukin-6 (IL-6) and -8 (IL-8), which, in turn, may reactivate a pro-invasive and GSC transcriptional programs, leading GBM cells to the “go” state, allowing them to migrate away from cytotoxic niches towards a supportive microenvironment (69, 82).

Decorin (DCN), a member of the small leucine-rich proteoglycans (PGs) family, has a vital role in the hypoxia-dependent activation of autophagy and anti-glioma therapy resistance, mainly due to their binding to VEGFR2 expressed by vECs, particularly in PNNs of glioma samples, or with the binding to c-Met and EGFR receptors expressed by GBM cells (83, 84). High levels of c-Met or DCN correlate with shorter progression-free survival (PFS) and overall survival (OS) in patients with GBM (85–88). The high-affinity DCN/receptor interaction leads to increased expression of paternally expressed gene 3 (Peg3), that physically associates with BECN1, recruiting LC3 into autophagosomes (89, 90). Complementarily, in GSC-enriched environments, GBM cells produce a high amount of PGs, such as DCN and Lumican, promoting chemotherapy resistance and cell survival (91). Curiously, as observed in several types of non-central nervous system tumors (92, 93), soluble DCN potently induces autophagy in GBM cells and contributes to an impairment of GBM cell migration *in vitro* experiments (94). Other extracellular matrix (ECM) proteins, such as endostatin, perlecan, and endorepellin, can influence tumor progression by regulating autophagy levels in endothelial cells, controlling vessel formation and neo-angiogenesis in response to hypoxia (95, 96).

## Autophagy Activation as a Response to Physiological Signals

Once PNN and other stress signals reactivate migration programs to drive plasticity and invasiveness in GBM cells, invasive growth along specific anatomical structures, especially at the vasculature and white matter tracts, is regarded as the main cause of poor therapeutic outcome of patients with GBMs. The migration occurs at perivascular niches (PVN), besides PNN, and considered the preferred and fastest route for GBM cell invasion through brain tissue (97). PVNs are fluid-filled spaces, continuous to the subarachnoid space, surrounding all blood vessels in the brain, including capillaries and arterioles. Based on histological information and *in situ* experiments, a widely accepted idea is that GBM cells actively seek out PVNs and migrate along with them (98, 99). For example, bradykinin, produced by cerebral vascular endothelial cells (vEC), acts as a strong chemotactic signaling peptide, guiding GBM cells toward PVN. Therefore, when injected into mice brain, the vast majority (over 85%) of human GBM cells move into contact with a blood vessel (100). At PVN, cerebral vECs are in the closest proximity to tumor cells. This heterotypic interaction induces a GSC transdifferentiation, which is critical for the malignant traits of the disease and supports the notion that stemness is a temporary reversible trait of GBM cells. The GSC phenotype is maintained by vECs *via* mediators, such as nitric oxide (NO), cyclic guanosine monophosphate (cGMP), and Notch1 ligands (97, 101–104). The stemness phenotype has been recently associated to autophagy activation and is one of the most important processes in the PVN responsible for the maintenance of GSC status besides PNN (105, 106). Additionally, the interaction between GBM cells and pericytes at PVN leads to chaperone-mediated autophagy in normal pericytes, building an immunosuppressed microenvironment that induces GSC phenotype and tumor growth (107). Interestingly, activation of protective autophagy in cerebral vECs is one of the essential physiological processes responsible for maintaining vascular homeostasis, and playing an important role in vECs proliferation, migration, and tube formation (108, 109). Other types of vEC-derived molecules also promote autophagy and correlate with stemness in GBM cells. For example, osteopontin (OPN), derived from the vEC, plays an oncogenic role and initiates a stem-promoting cascade and enhances autophagy through an integrin-CD44 dependent activation of HIF genes at PVNs (110, 111). OPN-elicited autophagy could promote cancer cell survival, resistance to chemotherapy drugs, and has been associated with increased glioma grade and migratory potential (112).

The melanoma-differentiation associated protein 9 (MDA-9, also called Syntenin-1) is another ECM protein that sponsors tumor invasion mainly by regulating the cell surface receptor Syndecan (113). In GBM, MDA-9 expression is an important regulator of cell invasion (114), stemness phenotype, and survival of GSCs through STAT3 and Notch1 pathways, respectively (115). Interestingly, the MDA-9 is responsible for activating protective autophagy in GSCs *in vitro* through the EGFR/FAK and EGFR/PKC axis, inhibiting anoikis (a suspension-induced form of apoptosis) by the hyperphosphorylation of Bcl-2 (116). In this scenario, autophagy often is activated in these cells as a compensatory pro-survival adaptation to detachment stress. In such cases, autophagy precedes (and usually avoids) anoikis by removing pro-apoptotic proteins in the cytosol. For example, depletion of ATG5 or ATG7 inhibits detachment-induced autophagy and enhances anoikis (117, 118). A higher expression of MDA-9 has been linked to higher glioma grade and short-term survival (119).

## Autophagy Activation as a Response to Anti-Glioma Therapies

RT plus concomitant and maintenance TMZ is the gold standard treatment and represent a major advance in the field of therapy for high-grade gliomas (7, 120). The addition of BVZ to standard treatment revealed an improvement in progression-free interval but had no effect on OS (121). Intriguingly, virtually all glioma therapies, including RT, TMZ and/or BVZ, are stronger inducers of autophagy pathway: several pre-clinical and clinicopathological studies indicate that increased autophagy activity help to desensitize GBM cells to treatment and it is linked with poor prognosis in different cancers (21). Inversely, others observations shown that excessive intensification of autophagic process lead to cell exhaustion and death (26, 62, 72, 122). So, despite the potential ‘dual’ role of autophagy has been clearly observed in cell-based studies, in ‘real’ disease, the predominant data conduct to the idea that therapy-induced autophagy is acting as an adaptive response and a protective mechanism in GBM cells instead of eliciting cell death.

The study of Natsumeda et al. (2011) is probably the first to show the induction of autophagy by TMZ in glioma cells and in reactive astrocytes of glioma patients by immunohistochemical analysis, indicating some type of stress response in tumor and normal cells (22). The addition of chloroquine (CQ) and its derivative hydroxychloroquine (HCQ) – both inhibitors of autophagy by blocking autophagosome fusion and degradation - to TMZ-treated glioma cells attenuates autophagy flux, induces accumulation of the proautophagy proteins (LC3-II) and promotes endoplasmic reticulum stress and cleavage of PARP (a marker of apoptosis) (123). Many other studies observed that blocking autophagosome formation enhances TMZ cytotoxicity, indicating that the autophagy pathway may protect GBM cells from TMZ-induced cytotoxicity (25, 123). For example, it has been demonstrated that CQ plus TMZ significantly increased the amounts of cleaved PARP (a marker for apoptosis) over those cells treated with TMZ alone. The pharmacological inhibition of autophagy by CQ also negatively dictates the migratory capacity of GBM cells, corroborating the role of autophagy with other aspects of adaptive phenotype and cell plasticity (124). While other authors have suggested that autophagy is the main component of TMZ-induced cytotoxicity and that inhibition of the autophagy significantly influences the antitumor effect of TMZ *in vitro* (20).

Ionizing radiation is the gold-standard adjuvant treatment for GBM. Radiotherapy also results in enhanced autophagy in GBM cells *in vitro* (125). When irradiated, many GBM cells undergo cell death by apoptosis, whereas GBM cells that do not undergo apoptosis activate autophagy, suggesting a protective mechanism (24, 125).

It has also been demonstrated that CQ treatments can increase radiosensitivity in GBM cells (25). Moreover, CQ worked synergistically with radiotherapy for induction of apoptosis in GSC; thereby acting as a protective mechanism (126). Another study showed that DNA-protein kinase-deficient GBM cells (DNA-PK), an enzyme that plays a critical role in DNA double-strand breaks repair, underwent massive ACD even after low doses of **γ-**radiation in cell lines *in vitro*. Intact DNA-PK pathway prevented ACD, but cells still exhibited a low apoptotic tendency, indicating that genetic background takes a leading role on the sensitivity of treatment and cell fate determination (127).

Another example of therapy-induced autophagy occurs after the use of antiangiogenic therapies in GBM. The addition of BVZ to conventional chemoradiation improved the PFS but did not affect OS (121). At the TME level, BVZ induces a hypoxic niche that results in protective autophagy sponsoring GBM cell resistance and survival. Alternatively, BVZ induced autophagy directly in GBM cells by suppressing the Akt-mTOR signaling pathway (128). Furthermore, BVZ-mediated autophagy is also dependent on interferon regulatory factor 1 (IRF1) expression in gliomas (129). Moreover, GBM cells expressing the stem cell markers CD133 and Sox2, and residing in the PVN, internalize BVZ through micropinocytosis, leading to autophagy activation and cell survival (130). Autophagy inhibition by ATG7 silencing rescued GBM sensitivity to BVZ treatments (131).

## Autophagy Activation as a Response to Internal Stimuli

Autophagy in GBM cells is triggered in response to external or internal stimuli. Internal stimuli is manifested directly by alterations in ATG or indirectly by oncogenic proteins commonly found aberrantly expressed in GBM and lower-grade gliomas. The following subsections cover the most important genetic events for gliomagenesis and their specific genetic aberrations associated with autophagy activation.

There are 16 known ATG in humans, four of which (ATG2B, ATG5, ATG9B and ATG12) are frequently mutated in gastric and colorectal cancers, and in hepatocellular carcinoma, and may be causally associated with cancer development by deregulating the autophagy process (132, 133). Large-scale genomic analysis indicates that core autophagy genes are generally not mutated in patients of 11 human cancers, including GBM, suggesting that the autophagy machinery is functional in cancer types investigated (134, 135). At a clinical perspective, several ATG signatures have been emerging as important prognostic factors for GBM patients, and autophagy high scores have been related to worse outcomes (136–138). For example, Wang and colleagues described that a robust 14-mRNA prognostic signature was an independent prognostic factor associated with OS in GBM’s patients (HR=1.9, 95% CI = 1.013-3.644, p value = 0.045) (136). Moreover, several other research groups have correlated the higher expression of ATGs with glioma aggressiveness, including patient’s poor survival and tumor progression (139–143). Despite their prognostic relevance, for future clinical applications, it is also important to integrate with other types of signatures (such as protein signatures).

Large-scale genomic studies showed that primary GBM arises from defects in three main molecular signaling pathways involving p53, Rb, and phosphoinositide 3-kinase (PI3K) (144). The phosphatidylinositol 3-kinase (PI3K)/Akt/mammalian target of rapamycin (mTOR) cascade is recognized as an important sensor of nutrient/growth factor availability and a major pathway regulating autophagy in human cancers. In a permissive microenvironment, active PI3K/Akt/mTOR cascade constitutively suppresses autophagosomes biogenesis by inactivating the ATG1/ULK1 complex or by sequestration and inactivation of BECN1, both considered key initiators of the autophagic pathway (145, 146). Inhibitors of Akt/mTOR activity, such as rapamycin analogs, intensify the autophagic process (147). However, under stressful conditions, PI3K/Akt/mTOR cascade is normally inactivated through extracellular signals, like intermittent hypoxia and depletion of nutrients, leading to the extrinsic activation of protective autophagy. Nevertheless, in GBM samples, activation of PI3K/Akt/mTOR cascade is observed in almost 90% of the cases, and caused by the overexpression of upstream activators, like epidermal growth factor receptor (EGFR) or c-Met, activating mutations of PI3CA (p110) or PIK3R1 (P85) (148–150), and inactivating mutations in the phosphatase and tensin homolog (PTEN), a negative regulator of PI3K activity (loss-of-function mutations in PTEN are present in almost 60-85% of GBMs) (151, 152). Moreover, the use of a potent PI3K inhibitor promotes autophagy activation at the expense of invasion and angiogenesis impairment in GBM cells. Furthermore, PI3K inhibition also restrained tumor growth and significantly prolonged mouse survival (153). In addition, GBM cells harboring mTOR hyper-activation, showed an increment of autophagy after the use of rapamycin (154).

The deregulation of the tumor protein p53 (TP53) pathway accounts for approximately 85% of GBMs, including alterations on CDKN2A, MDM2 and TP53 genes (155). Members of this signaling pathway have been described as modulators of migration, invasion, proliferation, and stemness, leading to poor prognosis in GBM patients (155). Regarding autophagy activation, nuclear p53 induces the expression of the ATGs: DRAM, and Sestrins 1/2. Indeed, DRAM1 is considered the regulator of the autophagy activation mediated by nuclear p53 (156–158), promoting migration and invasion of glioma stem cells (141). Interestingly, cytoplasmic p53 inhibits autophagy, but external stressors, such as nutrition starvation, induces the destruction of cytoplasmic p53, sustaining autophagy activation (159, 160). More recently, it has been shown that combined therapy with TMZ and CQ synergistically reduces cell proliferation and enhances apoptosis in p53-wild type cells. Overexpression of mutant p53 abolishes the autophagic vacuoles (161).

The Retinoblastoma gene (RB1) is a tumor suppressor gene commonly mutated or deleted in GBM and correlated with lower survival rates in astrocytomas patients (162, 163). Functionally, Rb inhibits cell cycle progression and promotes cell survival by controlling the function of the E2F transcription factor (164). Besides cell cycle transition control, Rb also influences tumor cell differentiation, senescence, apoptosis, and autophagy (165). Indeed, Rb downstream effector E2F1 directly mediates the expression of the autophagy-related genes LC3, ATG1, and DRAM (166). In GBM cells, it has been shown that Rb binds to E2F, repressing its activity, and leading to autophagy induction. Indeed, Rb activity or E2F1 silencing induced autophagic flux through increased autophagosome formation (167). Interestingly, while the binding of Rb to E2F promotes the activation of autophagy, Rb phosphorylation represses its binding to E2F and leads to apoptosis activation (168). In this scenario, it has already been shown that the Rb-E2F axis regulates the expression of the BNIP3 gene, an essential gene that mediates hypoxia-induced autophagy, promoting autophagosome formation in nutrient-deficient environments (169). Rb-induced autophagy is considered a resistance mechanism in GBM cells treated with etoposide or cisplatin (170, 171).

The most relevant and frequent oncogenic alterations in GBM patients involve the Epidermal growth factor receptor (EGFR), comprising 57% of patients. These alterations include mutations, rearrangements, amplifications, and splicing variants that lead to enhanced tumor growth, angiogenesis, survival, and stemness (148, 172). Intriguingly, due to the functional impact of EGFR alterations on tumor aggressiveness, lower- grade gliomas harboring EGFR amplification are considered “GBM-like tumors” due their aggressive phenotypic behavior (173). Beyond the known pathological role of EGFR on GBMs, their functions in autophagy regulation are emerging, indicating that it directly acts as a controller of the autophagic flux by mTOR signaling modulation (174, 175). EGFR-mediated autophagy exerts relevant roles in gliomagenesis, tumor progression, and therapy resistance (176). Clinically, GBM patients with low levels of EGFR and high expression of BECN1 have a median overall survival of 30 months, presenting a favorable response to radiotherapy (177). Therapeutically, the combination of tyrosine kinase inhibitors (TKI), such as erlotinib, with CQ increases the antineoplastic effect of the TKI on apoptosis-resistant GBM cells (178). Surprisingly, another EGFR inhibitor, called BIBU, impaired Akt and STAT3 activation, induced apoptosis death, and activated protective autophagy (179). The constitutively active mutant allele of EGFR, known as EGFRvIII is an important mediator of autophagy (180). It occurs in 20–30% of all human GBM, making it the most common EGFR mutant in GBM (181, 182). EGFRvIII- expressing GBMs are intrinsically resistant to apoptosis induced by radio- and chemotherapy (183, 184). Interestingly, these tumors have autophagy over-activation under hypoxic conditions and patients benefit from the use of CQ (180). Intriguingly, GBM cells harboring EGFRvIII alterations are more sensitive to the pharmacological inhibition of mTOR (185).

c-MET (also called HGFR) is a type of Receptor Tyrosine Kinase mutated in 6% and amplified in 4% of patients with GBM, leading to constitutive activity. Patients harboring c-MET gain-of-function alterations present a shorter survival and poor response to treatment (186). The enhancement of c-Met activity induces GBM cell survival, proliferation, invasion, angiogenesis, and stemness (187). The intracellular pathway triggered by c-MET is PI3K/Akt signaling. Additionally, cell invasion mediated by c-MET relies on Focal Adhesion Kinase (FAK) activity (188). Interestingly, c-MET expression was correlated with autophagy activation in GSCs, positively regulating their migratory and invasive capacity (141). c-MET expression abrogation by epigenetic silencing in glioma cells suppresses Akt pathway activation and up-regulates the expression of the autophagy-related protein Atg5, resulting in tumor growth reduction (189).

Isocitrate dehydrogenase 1 and 2 (IDH1/2) mutations are the most important molecular markers in diffuse gliomas due to their high impact on patient survival improvement and tumor development (190, 191). IDH1 mutations (IDH^mut^) are present in more than 80% of low-grade gliomas (grades II-III) and in secondary GBMs, but are rare in primary GBMs (190, 192). Mutations in IDH2 have been found in fewer than 3% of glial tumors. Patients with lower-grade gliomas (grades II-III) and glioblastoma show significantly longer OS in the presence of IDH1 or IDH2 mutations (192). The prognostic importance of IDH mutation is independent of other known prognostic factors, including age, grade, and MGMT methylation status. IDH mutations promote a metabolic reprogramming mainly due to the accumulation of the oncometabolite 2- hydroxyglutarate (2-HG), which, in turn, induces the epigenetic silencing of several genes from the glycolytic pathway (193, 194). Moreover, IDH1^mut^ is associated with a distinct hypoxia/angiogenesis transcriptome signature and stabilization of HIF-1a levels in glioma cells (195), important autophagy regulators (see above). Recently, four different groups identified distinct autophagy signatures with prognostic value in GBMs. High autophagy risk signatures were correlated with patients’ worse outcomes. Besides the absence of gene intersection between the signatures, all four achieve the same results: patients with IDH^mut^ tumors presented a lower autophagy-related risk signature compared to IDH wild-type (IDH^wt^) gliomas, denoting an increased autophagy activation in IDH^wt^ GBMs (136, 137, 196, 197). In the same direction, beyond the gene signatures, it has been shown that higher expression levels of the ATG proteins: LC3, Beclin-1, and p62 are more prevalent in IDH^wt^ gliomas in than IDH^mut^ gliomas (139).

Promoter methylation of the O-6-Methylguanine-DNA Methyltransferase (MGMT) gene is a prognostic marker in patients with glioma because MGMT methylation leads to better response to alkylating agents, such as TMZ (198). Indeed, patients harboring MGMT-methylated GBMs had a 10-month and 4-month higher median overall and PFS, respectively, compared with MGMT-non-methylated patients (199). Interestingly, two different groups showed that MGMT-methylated gliomas presented a lower autophagy risk score compared with MGMT-non-methylated patients (136, 137). In agreement with these data, GBM cell lines that naturally do not express MGMT, highly activate autophagy after TMZ treatment. However, when cells were stably transfected with MGMT, the number of autophagic vacuoles was abrogated after TMZ treatment (122).

Finally, beyond the role of oncogenes, tumor suppressor genes and their downstream signaling molecules, the control of autophagy activation in GBM also relies on the signaling pathways involved in stemness (200). These pathways are mainly involved in the acquisition and maintenance of the GSC phenotype, including Notch, Wnt/β-catenin, and Hedgehog pathways. In gliomas, the activation of *Notch signaling* correlates with more aggressive tumor phenotypes (201). Besides activation of the Notch pathway, its members and ligands are rarely mutated in GBMs (202). The connection between the Notch pathway and autophagy was first described in U87MG and U251 GBM cell lines. When the Notch1 receptor was genetically silenced in these cells, they showed reduced proliferation and viability. GBM growth impairment was correlated with the augmented expression of the autophagy-related proteins Beclin-1 and LC3-II in NOTCH1-silenced cells (203). Complementarily, when autophagy was induced in GSCs by mTOR inhibition, the Notch1 receptor was degraded. Indeed, the impairment of Notch1 signaling induced by autophagy activation led to a decreased tumorigenicity and self-renewal capacity of GSCs (204). Interestingly, the degradation of Notch1 by autophagy is mediated *via* autophagosome-precursor vesicles positively expressing the autophagy-related protein ATG16L1 (205), and by the direct binding of p62 to Notch1 Intracellular Domain (NICD) (206). In contrast, the pharmacological blockage of Notch1 induces cytoprotective autophagy in GBM cells. However, when these cells were exposed to the combination of Notch1 and autophagy inhibitors, treatment resistance was overcome, thus augmenting apoptotic cell death (207). Interestingly, Notch1 signals can be regulated by autophagy activation *via* ATG16L1-positive autophagosomes, modulating stem cell development, and neurogenesis (205).

*Wnt signaling* plays a critical role in GSC phenotype, therapeutic resistance and invasiveness (208, 209). Mutations in the members of Wnt signaling pathway are not common, but epigenetic alterations are frequently observed in GBMs (210). The inhibition of Wnt signaling by the IWR-1 inhibitor leads to an augment of the expression of the autophagy-related proteins, LC3-II, and Beclin-1 (211). Complementarily, another group showed that the silencing of the intracellular players of Wnt signaling, TCF4, and CTNNB1/β-catenin, induced the up-regulation of SQSTM1/p62, increasing the autophagy flux. Interestingly, Wnt pathway inhibition sensitizes GBM cells to autophagy inhibition with CQ (212). Regarding chemotherapy response to TMZ, the blocking of the Wnt/β -catenin pathway by the activity of the DAB2IP protein is responsible for TMZ resistance through the expression of the autophagy-related protein ATG9B. Interestingly, the combination of TMZ with a Wnt signaling inhibitor can overcome this resistance (213). Indeed, autophagy activation mediated by nutrient starvation in GBM cells down-regulates several mediators Wnt signaling, including activated β-catenin (214).

*Hedgehog (Hh) signaling* enhances the migratory and invasive capacity of cells through the activation of PI3K/Akt pathway in GBM cells (215). Moreover, it stimulates the growth and tumorigenicity of gliomas, mainly by controlling stemness status (216). Besides the lack of mutations on Hh pathway components in GBMs, it has been shown that the glioma-associated oncogene homolog 1 (GLI1) zinc-finger transcription factors, terminal effectors of the Hh pathway, presents two tumor-specific splicing isoforms, which directly influences tumor malignancy (217). Intriguingly, the activation of Hh signaling is correlated with the modulation of autophagy in several cancer types. Indeed, the inhibition of the Hh pathway negatively controls tumor proliferation by activating autophagy (218). In GBM cells, the use of GANT-61, a specific inhibitor of Gli1 and Gli2, activates autophagy, inducing LC3-II expression, and by negatively modulating the expression of stemness markers and tumor proliferation (219). Pharmacologically, the use of GANT-61 enhances the cytotoxic effect of TMZ by the increment of acid vesicles and Beclin-1 expression (220). Furthermore, the regulatory domain of PTCH1, the main receptor of the Hedgehog pathway, interacts physically with the autophagy-related protein ATG101 in a nutrient starvation microenvironment, inhibiting the autophagic process (221). Additionally, GBM cells overexpressing the stem marker SOX3 showed an upregulation in Hh pathway activity and suppression of autophagy, leading to an increment in proliferation and invasion (222).

## The Last Frontier: The Therapeutic Potential of Autophagy Inhibitors for the Treatment of GBM

**Figure 1:**
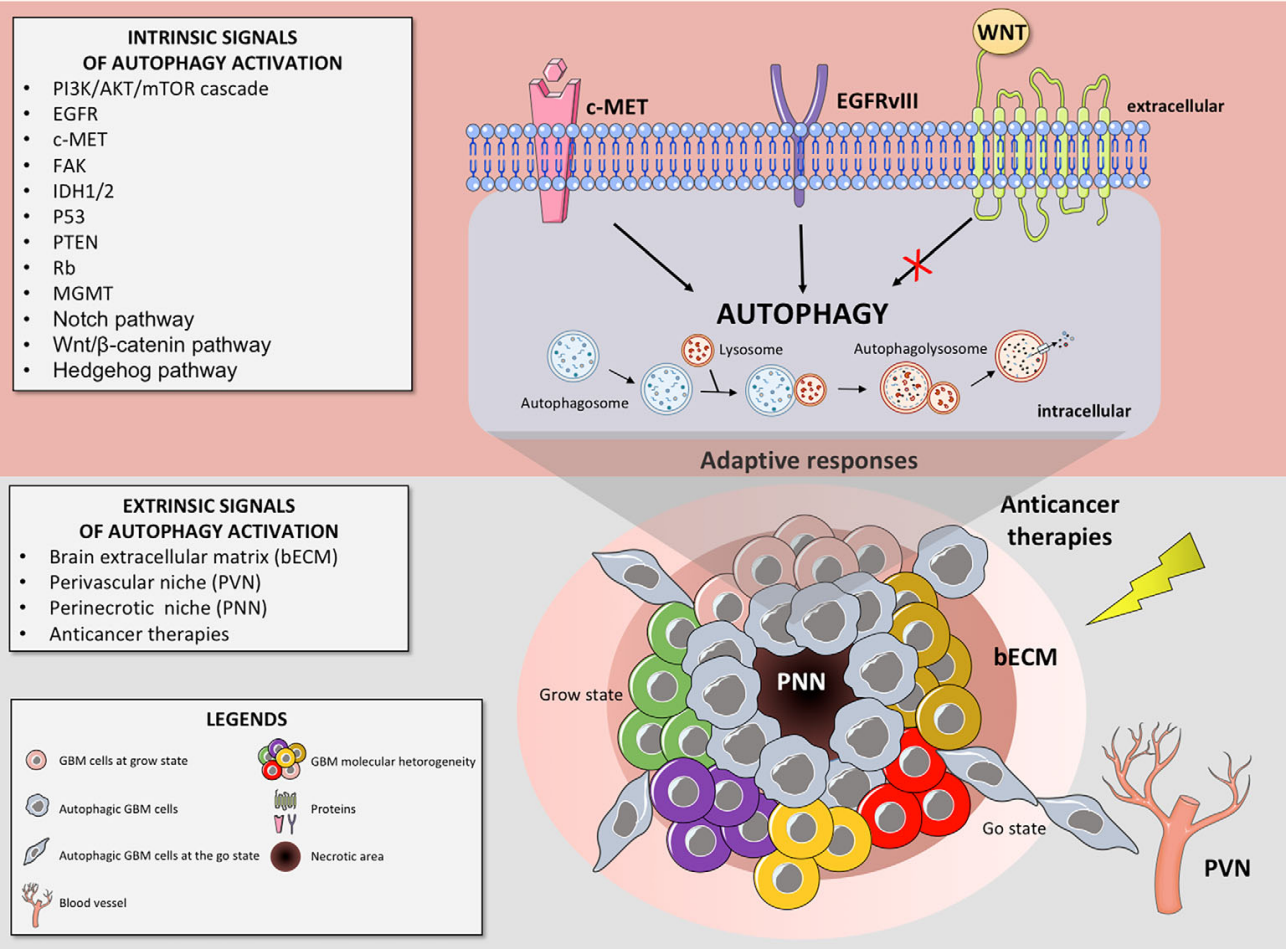
Autophagy can be triggered by intrinsic and/or extrinsic GBM cells signals, contributing to tumor cell proliferation (grow state), invasion (go state) and resistance to therapies. Thus, autophagy may function as a mechanism of tumor cell survival and progression in a hostile microenvironment. The intrinsic signals activating autophagy in GBM consist of specific gene expression levels alterations (like in the cMET gene), mutations (like the mutation in the EGFR gene that gives rise to the active mutant EGFRvIII) and/or specific signaling pathways perturbations (such as in the Wnt pathway). The extrinsic signals associated with autophagy activation in GBM cells are: 1) the perinecrotic niches (PNN), composed by highly proliferative GBM cells and where the autophagy activation may function as a cellular adaptive response to hypoxia; 2) the perivascular niche (PVN), where the vascular endothelial cells can interact with the GBM ones, inducing autophagy and a stemness phenotype of those tumor cells; 3) the brain extracellular matrix (bECM), whose components may regulate autophagy; and 4) the anticancer therapies, which can activate autophagy as a cytoprotective mechanism.

GBM retains a poor prognostic value and remains incurable. Despite our growing understanding of the mechanisms underlying drug resistance, the standard therapy has not changed over the last 16 years (7). Up to date, no new therapies improve OS when added to standard therapy, with an exception for the recent Tumor Treating Fields (TTFields) in GBM (223). As we see above, under pathophysiological circumstances, autophagy is a key driver of GBM resistance, allowing cellular adaptive survival towards extrinsic (e.g. hypoxia, drugs or ionizing radiation) or intrinsic (genetic aberrations) stress stimuli (**Figure 1**). Accordingly, from a standpoint of treating GBM disease, targeting autophagy emerge as a new potential therapy and it has been considered a potential candidate to improve the treatment of patients with GBM (224, 225). In this scenario, the use of the autophagy inhibitors, such as CQ or HCQ, has been explored in clinical studies. Those trials mainly focused on the therapeutic potential of autophagy inhibition combined to standard therapies for GBM patients.

A small phase III trial observed a median overall survival of 24 months for patients treated with CQ plus conventional therapy (i.e. surgery, radiotherapy, and carmustine-based chemotherapy) compared to 11 months for patients treated with conventional therapy (226). In a single institutional study with 123 patients, the same authors showed that the addition of CQ to surgery, radiotherapy, and carmustine-based chemotherapy consistently exerts an adjuvant effect, adding more than 13 months in patients’ median survival in comparison to control patients (227). However, despite the favorable results in GBM patients treated with CQ combined to surgery, radiotherapy, and carmustine, a phase I/II trial combining HCQ with radiotherapy and TMZ-based chemotherapy showed that the maximum tolerated dose of HCQ was unable to consistently inhibit autophagy and showed no improvement in patient OS (228). To transpose those issues, a new phase I/II trial (NCT02432417) was designed to compare patients treated with concurrent ionizing radiation and TMZ- based chemotherapy with patients treated with this combination plus a most appropriate CQ dose. Indeed, recent data published by the group showed that 200 mg of CQ is a feasible dose to use in those patients, since 400 mg of CQ induced several severe adverse events. Moreover, preliminary data analysis showed an improvement of more than 9 months in the OS of GBM patients harboring EGFRvIII alterations compared with patients without this genetic variant (229). The International Cooperative Phase III Trial is an active clinical trial that evaluates the use of CQ or Valproic acid as an adjuvant to conventional therapy in high-grade gliomas (NCT03243461).

Due to these positive results in the early clinical trials, it is essential to invest in studies to evaluate CQ effects on GBM patients’ survival using larger cohorts. Moreover, it is also necessary to determine HCQ efficacy and tolerated doses and invest in discovering new drugs with similar action mechanisms.

## Future Directions: Crossing the Valley of Death

The discrepancy between pro- and anti-tumor functions of autophagy, modulating GBM cell plasticity or alternative mechanism of cell death, emphasizes a question that has emerged as critical in translational science: how wide gap exists between basic and clinical biomedical data? The establishment of interdisciplinary research institutes stimulating collaborations between clinicians, physician-scientists, and basic biologists are critical to bring these areas together, but the importance of the critical interpretive reviews of literature data is also fundamental.

By examining carefully the literature we realize that explanations for the controversies of whether the autophagy pathway promotes survival or death are still elusive. Sometimes the balance between autophagic-dependent pro-survival or pro-death signals depends greatly on the quantitative relationship between them: over to moderate level of autophagy activation is cytoprotective, whereas high levels of autophagy are cytotoxic. Sometimes there are even conflicting reports with the same drug treatment in the same experimental model. Pre-clinical and clinical data indicate that autophagy is an emblematic example of a rescue pathway that contributes profoundly to a pro-tumoral adaptive response. On the other hand, high levels of activation lead to cytotoxic autophagy, which seems to be exclusively induced by excessive and homogeneous stress signals from *in vitro* cell-based studies.

From a standpoint of understanding the real GBM disease, the spatial and temporal heterogeneity of the external and internal stimuli must be considered. Steep gradients in pO2, pH, nutrient availability and drug perfusions ranging from pathological/therapeutic conditions to those found in normal tissues, added to the high levels of genetic intratumoral heterogeneity, are hallmark features of GBM. Thus, it implies that the levels of autophagy activation may also show extensive spatial heterogeneity in subpopulations from the same tumor determining divergent cell fates.

Despite the ability of many compounds, like CQ and HCQ, to inhibit autophagy and demonstrated good efficacy in preclinical studies, clinical trials for GBM continue showing no significant survival or clinical benefit, due to sparse anti-glioma activity or severe side effects. Thus, the last frontier to test the therapeutic potential of autophagy pathway in GBM awaits the development of compounds that can achieve more consistent inhibition.

Finally, only combinatorial therapy targeting autophagy with cytotoxic drugs in the adjuvant setting for GBM patients, associated with the development of less toxic and higher specific autophagy inhibitors, may inhibit adaptive response and enhance the sensibility of glioma cells to conventional therapies. In the context of an incurable human disease, pharmacological inhibition of autophagy would represent a promisor therapeutic target for radio- and chemosensitization of GBM cells.

## Author Contributions

EHFJ, MB, and LTI wrote the review and designed the figures. FBF and AAC critically revised the final version and ETC edited and supervised the whole work. All authors contributed to the article and approved the submitted version.

## Funding

This work was supported by grants from FAPESP (16/07463-4, 16/06857-9), Ludwig Institute for Cancer Research and Hospital Sírio-Libanês.

## Conflict of Interest

The authors declare that the research was conducted in the absence of any commercial or financial relationships that could be construed as a potential conflict of interest.
